# Latifolin, a Natural Flavonoid, Isolated from the Heartwood of *Dalbergia odorifera* Induces Bioactivities through Apoptosis, Autophagy, and Necroptosis in Human Oral Squamous Cell Carcinoma

**DOI:** 10.3390/ijms232113629

**Published:** 2022-11-07

**Authors:** Hyung-Mun Yun, Ji Eun Park, Joon Yeop Lee, Kyung-Ran Park

**Affiliations:** 1 Department of Oral and Maxillofacial Pathology, School of Dentistry, Kyung Hee University, Seoul 02447, Korea; 2 National Development Institute for Korean Medicine, Gyeongsan 38540, Korea; 3 Gwangju Center, Korea Basic Science Institute (KBSI), Gwangju 61751, Korea

**Keywords:** apoptosis, autophagy, *D. odorifera*, Latif, necroptosis, OSCCs

## Abstract

Oral squamous cell carcinoma (OSCC) is the most common malignant neoplasm with frequent metastasis and high mortality in the oral cavity. Plant-derived natural compounds are actively progressing as a trend for cancer treatment. Latifolin (Latif), is a natural flavonoid isolated from the heartwood of *Dalbergia odorifera* T. Chen (*D. odorifera*) has been known to have beneficial effects on anti-aging, anti-carcinogenic, anti-inflammatory, and cardio-protective activities. However, the anti-cancer effects of Latif are unknown in OSCC. Herein, as a result of analysis in terms of the aggressive features of OSCCs, we found that Latif significantly inhibited the cell proliferation of human YD-8 and YD-10B OSCCs, and caused the anti-metastatic activities by effectively blocking cell migration, invasion, and adhesion via the inactivation of focal adhesion kinase (FAK)/non-receptor tyrosine kinase (Src). Moreover, we found that Latif induced apoptotic cell death to suppress the cell survival and proliferation of YD-10B OSCCs by targeting PI3K/AKT/mTOR/p70S6K signaling. Finally, we analyzed in terms of autophagy and necroptosis, which are other mechanisms of programmed cell death and survival compared to apoptosis in YD-10B OSCCs. We found that Latif suppressed autophagic-related proteins and autophagosome formation, and also Latif inhibited necroptosis by dephosphorylating necroptosis-regulatory proteins (RIP1, RIP3, and MLKL). Given these findings, our results provided new evidence for Latif’s biological effect and mechanism in YD-10B OSCCs, suggesting that Latif may be a new candidate for patients with OSCCs.

## 1. Introduction

*Dalbergia odorifera* T. Chen (*D. odorifera*) belongs to the Leguminosae family. It is known as kangjinhyang in Korea, is a perennial tree that mainly grows in East Asian countries, and the heartwood extracts have been frequently used as traditional Chinese medicine (TCM) to treat various diseases, including blood disorders, diabetes, cancer, cardiovascular diseases, and ischemia [1,2]. The extracts and secondary metabolites of *D. odorifera* have also been reported to have anti-bacterial, anti-inflammatory, anti-osteoporotic, anti-osteosarcoma, and anti-oxidative activities [2,3,4]. Thus, the extracts and secondary metabolites of *D. odorifera* are considered valuable resources for novel drug development. A natural flavonoid, Latifolin (Latif), isolated from the heartwood of *D. odorifera* is an inducer of quinone reductase, a carcinogen-detoxifying enzyme in mouse hepatoma cells [5]. Latif has also been reported to have protective effects against oxidative stress-induced senescence through the upregulation of silent information regulator 1 in human dermal fibroblasts and anti-inflammatory effects through the upregulation of nuclear transcription factor E2-related factor 2 in primary peritoneal macrophage [6,7]. However, Latif’s anti-cancer effects and mechanisms in oral squamous cell carcinoma (OSCC) have not yet been reported.

OSCCs are the most common malignant tumors that invade the local tissues of the oral cavity [8,9]. The OSCCs exhibit aggressive features with rapid proliferation, frequent infiltration into adjacent tissues, and a high recurrence rate [10]. For this reason, OSCC seriously threatens the life of patients. Despite many efforts and improvements in diagnosis and treatment, the five-year survival rate of patients with OSCCs is less than 60% due to aggressive features such as metastasis, recurrence, and drug resistance [8,11]. Thus, there is a need for improved treatment strategies using bioactive compounds for the treatment and prevention of OSCC.

In the present study, we isolated a flavonoid compound, Latif (>100% purity), from the heartwood of *D. odorifera*, and the Latif was dissolved in a widely used solvent, dimethyl sulfoxide (DMSO). We investigated the biological effects, and underlying signaling mechanisms of Latif in human YD-8 and YD-10B OSCCs established from tongue cancer tissues of patients with OSCCs

## 2. Results

### 2.1. Identification and Isolation of Latif from Heartwood of D. odorifera

**Figure 1:**
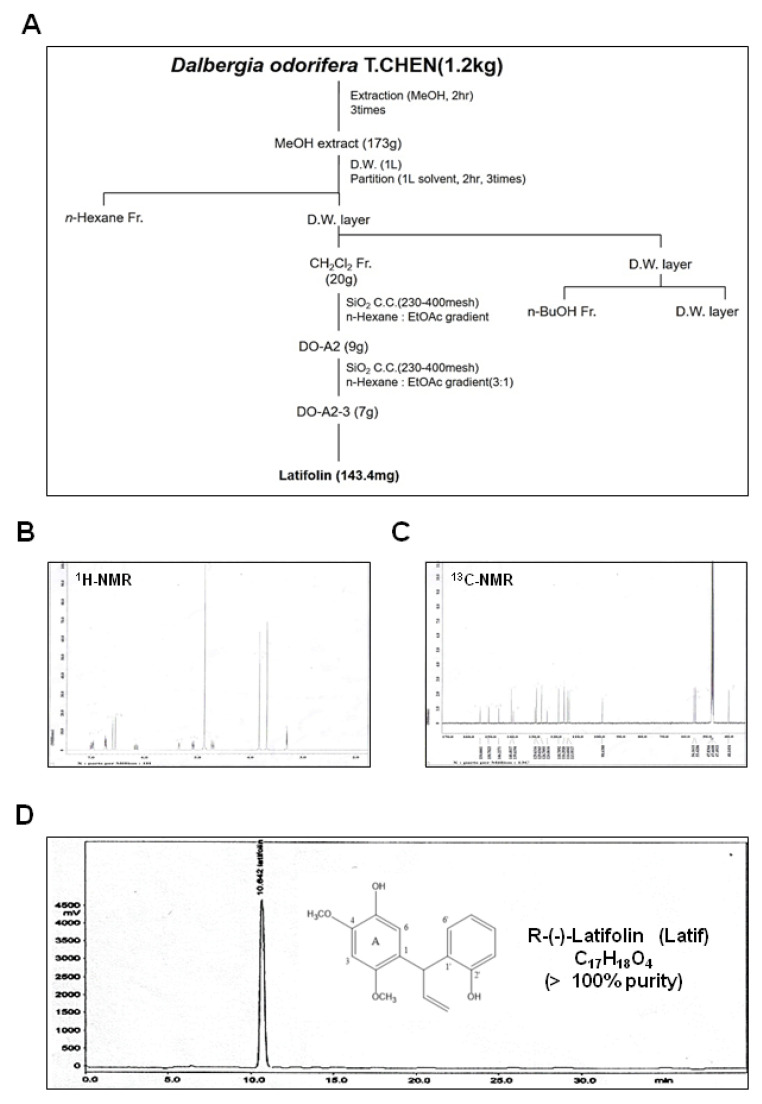
Isolation of Latif from the heartwood of *D. odorifera*. (**A**) Scheme of Latif isolated from the heartwood of *D. odorifera*. (**B**,**C**) The ^1^H-NMR (500 MHz, CD_3_OD) spectrum (**B**) and ^13^C-NMR (125 MHz, CD_3_OD) spectrum (**C**) of Latif. (**D**) The HPLC and chemical structure (inset) of Latif.

*D. odorifera* (1.2 kg) was extracted twice with MeOH (2 L, 2 h) by refluxing in a heating mantle. The crude extract (173 g) was suspended in 60% MeOH (1 L) and then solvent partitioned using n-Hexane (0.8 L *×* 2 times), CH_2_Cl_2_ (0.8 L *×* 2 times). The CH_2_Cl_2_ (20 g) was divided into six fractions through open-column chromatography (silica gel 200–300 mesh, n-Hexane:EtOAc gradient). The two fractions (9 g) were divided into four fractions through open column chromatography (silica gel 200–300 mesh, n-Hexane:Acetone = 3:1). The Latif compound (pale yellow powder, 143.4 mg) was obtained from three subfractions (7 g) (Figure 1A). The structure of Latif was identified by comparing the spectral data using before literature [12]. ^1^H-NMR (500 MHz, CD_3_OD) assign data: δ 3.84 (3H, s, -OCH_3_), 3.86 (3H, s, -OCH_3_), 4.71–5.35 (1H, m, =CH_2_ trans, -CH-CH=CH_2_, J = 17.0, 10.0, 6.0 and 1.6 Hz), 6.2–6.57 (1H, m, -CH=CH_2_, J = 17.0, 10.0 and 6.0 Hz), 6.52 (1H, s, 3-H), 6.78 (1H, s, 6-H), 6.85–7.19 (4H, m, B ring) (Figure 1B, and Supplementary Figure S1A). ^13^C-NMR (125 MHz, CD_3_OD) assign data: δ 40.1 (C-H_A_), 55.4 (2-OCH_3_), 56.1 (4-OCH_3_), 98.6 (C-3), 113.9 (=CH_2_), 114.6 (C-3′), 116.20 (C-6), 118.7 (C-5′), 124.0 (C-1), 126.7 (C-6′), 129.0 (C-4′), 129.6 (C-1′), 139.6 (C-5), 140.4 (C-Hx), 146.2 (C-4), 150.7 (C-2), 154.6 (C-2′) (Figure 1C and Supplementary Figure S1B). The HPLC chromatogram and chemical structure of Latif (>100% purity) are shown in Figure 1D.

### 2.2. Latif Inhibits Cell Proliferation in Human OSCCs

**Figure 2:**
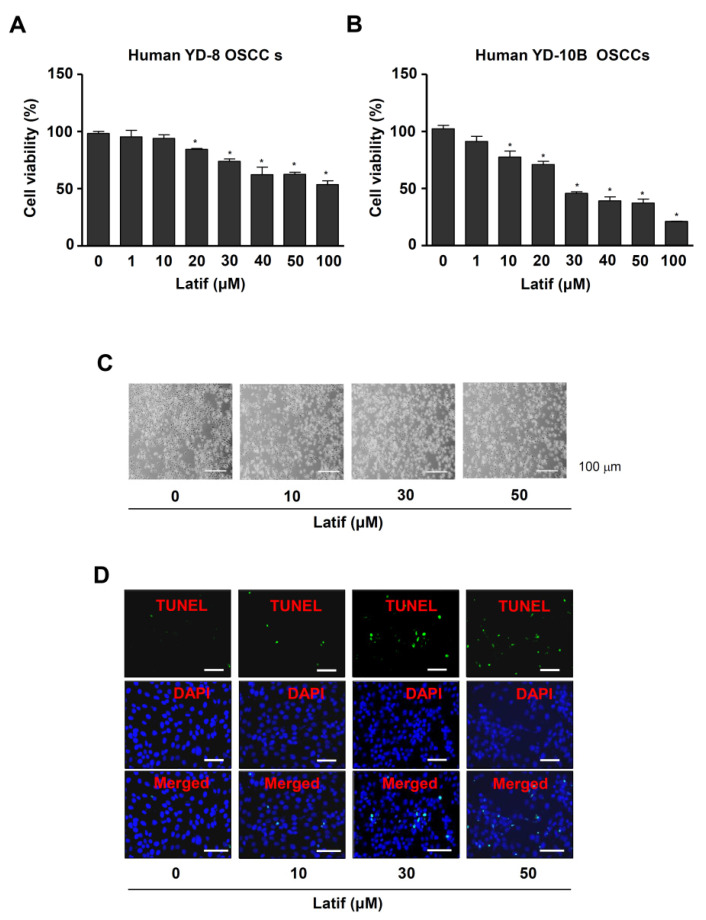
Effects of Latif on proliferation and apoptotic alteration in human OSCCs. (**A**,**B**) Cell viability was assessed after Latif treatment for 24 h at concentrations ranging from 0 to 100 μM in human YD-8 and YD-10B OSCCs using an MTT assay. (**C**) Amounts of 10, 30, and 50 μM Latif was treated for 24 h in YD-10B OSCCs, and apoptotic morphological changes were monitored under a light microscope. (**D**) Nuclear apoptotic DNA fragments were analyzed by TUNEL and DAPI staining and monitored under a fluorescence microscope. Scale bar: 50 μm. Data are represented as mean ± S.E.M. Significance was set at *p* < 0.05, indicated by an asterisk in the graph.

To investigate the anti-OSCC activities of Latif against the aggressive proliferation of OSCCs, we treated various concentrations (0, 1, 10, 20, 30, 40, 50 and 100 μM) in human YD-8 and YD-10B OSCCs. The MTT assay showed that Latif significantly suppressed cell proliferation in a dose-dependent manner in OSCCs, whereas Latif blocked cell proliferation more effectively in human YD-10B OSCCs compared to human YD-8 OSCCs (Figure 2A,B). Human YD-10B OSCCs were used for the next experiments, and Latif was treated at concentrations of 10, 30, and 50 μM.

### 2.3. Latif Induces Apoptotic Cell Death in Human YD-10B OSCCs

To find out whether Latif-mediated effects were associated with apoptotic cell death, we next examined morphologic changes and DNA fragmentation. YD-10B OSCCs were incubated with Latif for 24 h. The observation for apoptotic morphologic changes showed that YD-10B OSCCs decreased cell size, changed to a round shape and lost attachment on the surface compared to the control (Figure 2C). Moreover, the TUNEL assay exhibited that Latif induced apoptotic DNA fragmentation in the nucleus of YD-10B OSCCs compared to the control (Figure 2D). 

### 2.4. Latif Induces Apoptotic Caspase Cascade and Suppresses Cell Cycle Proteins in Human YD-10B OSCCs

**Figure 3:**
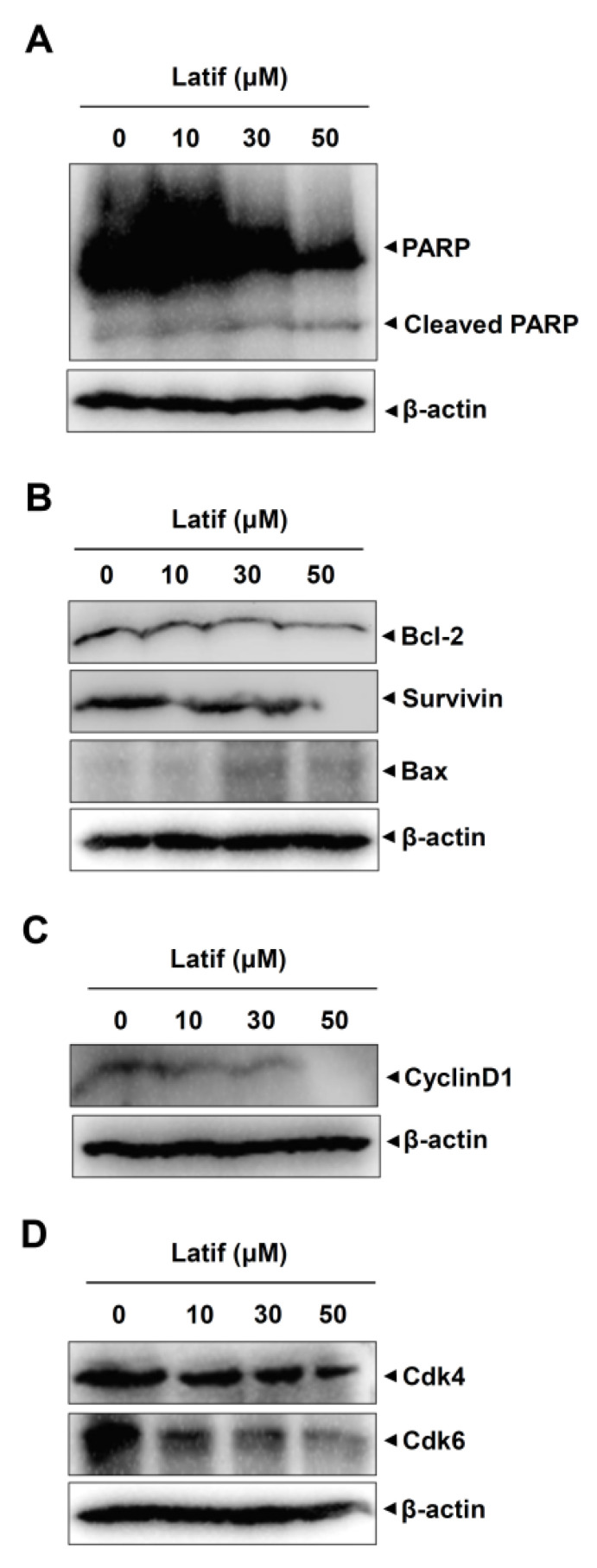
Effects of Latif on apoptosis- and cell cycle-related proteins in human YD-10B OSCCs. (**A**–**D**) Amounts of 10, 30, and 50 μM Latif was treated for 24 h in YD-10B OSCCs, equal amounts of proteins were obtained from total cell lysates, and then PARP, cleaved PARP (**A**), Bcl-2, Survivin, Bax (**B**), CyclinD1 (**C**), Cdk4, and Cdk6 (**D**) were analyzed using Western blot analysis. β-actin level was detected as a loading control. Data are representative of the results of three experiments.

Based on the cellular and molecular changes of YD-10B OSCCs, we validated apoptotic cell death by detecting caspase cascade using western blot analysis. Since PARP is a main apoptosis feature and is cleaved by active caspase-3, we first analyzed PARP cleavage in YD-10B OSCCs. The results showed that Latif increased PARP cleavage products, whereas the full-length PARP proteins were decreased in a dose-dependent manner (Figure 3A). We then analyzed the pro- and anti-apoptotic proteins that trigger and control the caspase cascade in YD-10B cells. As shown in Figure 3B, Latif reduced Bcl-2 and Survivin protein expressions, whereas increased Bax expression (Figure 3B). In addition, we analyzed cell cycle proteins that play central roles in regulating cell proliferation and death. The Western blot analysis showed that Latif reduced the expression levels of Cyclin D1 protein along with Cdk4 and Cdk6 proteins that form active complexes and promote cell proliferation (Figure 3C,D).

### 2.5. Latif Suppresses the AKT Signaling Pathways in Human YD-10B OSCCs

**Figure 4:**
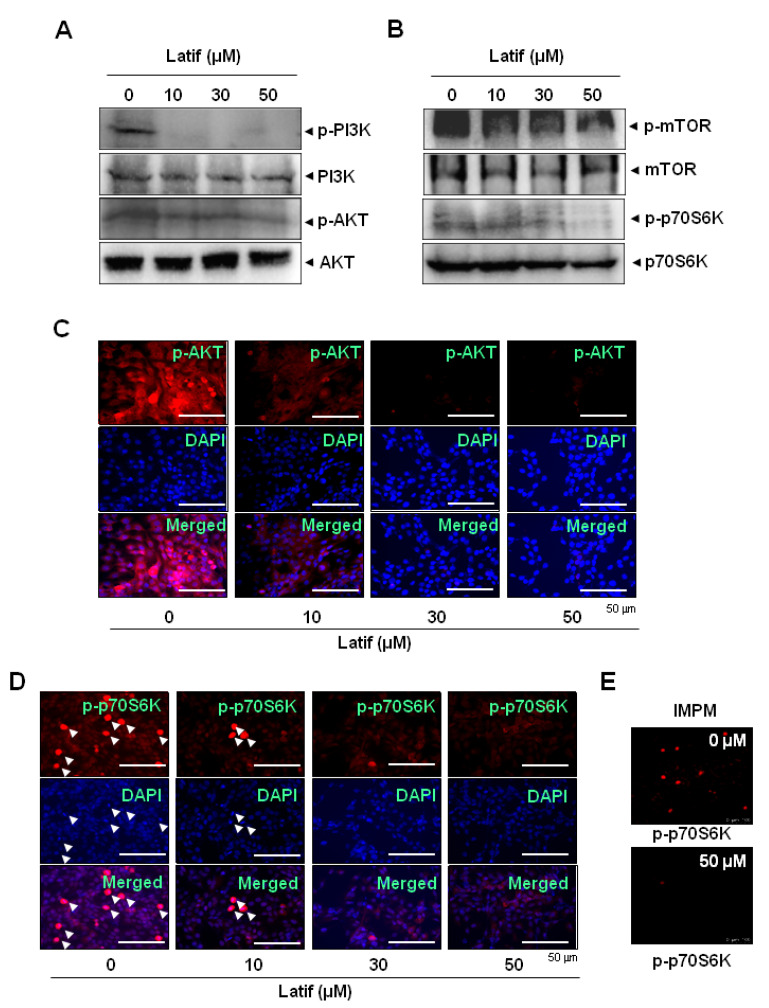
Effects of Latif on the AKT pathway in human YD-10B OSCCs. (**A**,**B**) Amounts of 10, 30, and 50 μM Latif was treated for 24 h in YD-10B OSCCs, and then phospho-PI3K (p-PI3K), PI3K, phospho-AKT (p-AKT), AKT (**A**), phospho-mTOR (p-mTOR), mTOR, phospho-p70S6K (p-p70S6K), and p70S6K (**B**) were analyzed using Western blot analysis. β-actin level was detected as a loading control. (**C**–**E**) The cellular expression of p-AKT (**C**) and p-p70S6K (**D**,**E**) was analyzed using an immunofluorescence assay and images were observed using a fluorescence microscope (**C**,**D**) and validated using the intravital multi-photon microscope system (IMPM) for p-p70S6K (**E**). DAPI (blue) was used to stain the nucleus. Arrowheads: p-p70S6K- and DAPI-positive cells. Data are representative of the results of three experiments.

To examine the cellular mechanism of Latif-induced apoptotic cell death, we focused on PI3K/protein kinase B (AKT)/mammalian target of rapamycin (mTOR)/p70S6K signaling since the pathway is frequently amplified in OSCCs and is important in therapeutic approaches [13,14]. The Western blot analysis showed that Latif induced the dephosphorylation of PI3K and AKT proteins regardless of total protein expressions in YD-10B OSCCs (Figure 4A). Subsequently, we found that Latif reduced the phosphorylation of mTOR and p70S6K proteins that are AKT downstream proteins in YD-10B OSCCs (Figure 4B). We further confirmed the effects of Latif on the AKT signaling pathway through fluorescence microscopy. The observation also showed decreases in the phosphorylation of AKT and p70S6K in YD-10B OSCCs (Figure 4C–E). More importantly, Latif reduced active cell division compared to the control, as observed in p-p70S6K- and DAPI-positive cells indicated by arrowheads (Figure 4D).

### 2.6. Latif Suppresses Autophagy- and Necroptosis-Regulating Proteins in Human YD-10B OSCCs

**Figure 5:**
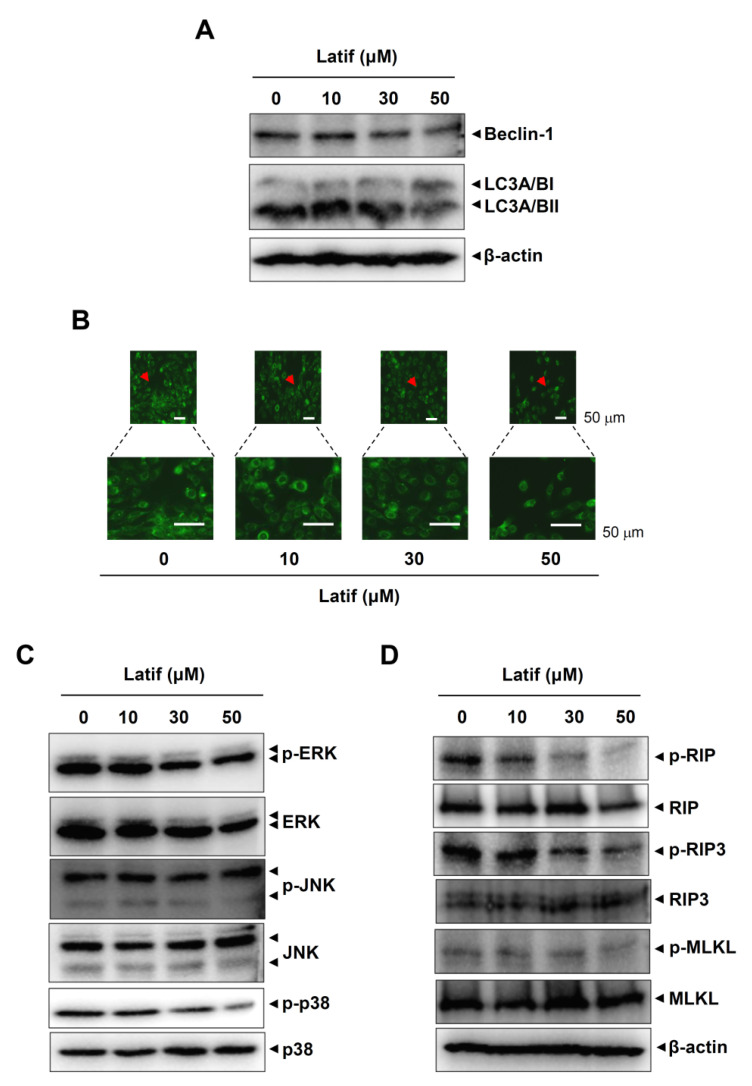
Effects of Latif on autophagy and necroptosis in human YD-10B OSCCs. (**A**) Amounts of 10, 30, and 50 μM Latif was treated for 24 h in YD-10B OSCCs, and then Beclin1, LC3A/BI, and LC3A/BII expressions were assessed using western blot analysis. (**B**) Cells were treated with 10, 30, and 50 μM Latif, and then DAPGreen-positive autophagosome was observed using a fluorescence microscope. Arrowheads: Magnfied region. (**C**) Phospho-ERK1/2 (p-ERK1/2), ERK1/2, phospho-JNK (p-JNK), JNK, phospho-p38 (p-p38), and p38 levels were assessed using western blot analysis. (**D**) The cells were treated with Latif, and phospho-RIP (p-RIP), RIP, phospho-RIP3 (p-RIP3), RIP3, phospho-MLKL (p-MLKL), and MLKL were analyzed using Western blot analysis. The β-actin level was detected as a loading control. Data are representative of the results of three experiments.

We next examined whether Latif affects other modes (autophagy and necroptosis) of cell survival and death in YD-10B OSCCs. First, we analyzed the molecular machinery of autophagy using Western blot analysis. The results showed that Latif decreased the expression levels of Beclin-1 and microtubule-associated protein light chain 3A/B (LC3A/B) (Figure 5A). We also monitored the formation of autophagic vacuoles in YD-10B OSCCs through fluorescence microscopy and found that Latif reduced the autophagosome formation in YD-10B OSCCs (Figure 5B). Since mitogen-activated protein kinases (MAPKs) are associated with autophagosome formation, we further examined whether MAPKs are involved in Latif-mediated autophagy. Western blot analysis showed that Latif effectively reduced p38 phosphorylation and slightly decreased ERK1/2 phosphorylation, whereas it did not affect JNK phosphorylation (Figure 5C). Next, we analyzed the molecular machinery of necroptosis in YD-10B OSCCs, and the results showed that Latif suppressed the phosphorylation levels of receptor-interacting serine/threonine-protein kinase (RIP)1, RIP3, and mixed lineage kinase domain-like pseudokinase (MLKL) (Figure 5D). Overall, our results suggest that Latif has anti-cancer effects on OSCCs by increasing apoptotic cell death and decreasing autophagy and necroptosis.

### 2.7. Latif Inhibits Cell Migration, Invasion, and Adhesion in Human YD-10B OSCCs

**Figure 6:**
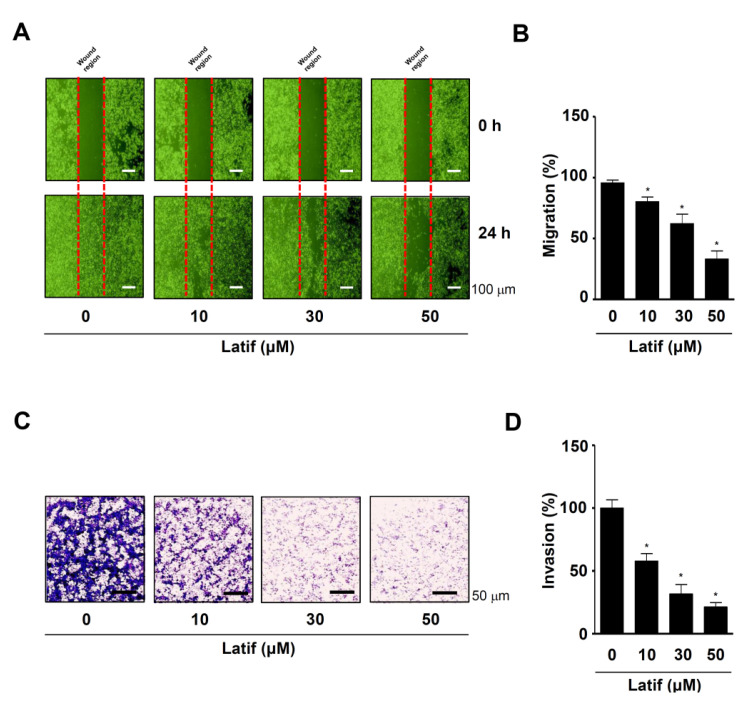
Effects of Latif on cell migration and invasion in human YD-10B OSCCs. (**A**,**B**) Amounts of 10, 30, and 50 μM Latif were treated for 24 h in YD-10B OSCCs, and then cell migration was then analyzed using a wound-healing assay. At the indicated time points, the migration rates were monitored using a light microscope (**A**). The bar graph shows the relative value of migration (%) normalized to the control (**B**). (**C**,**D**) The invasion rates of Latif-treated cells (10, 30, and 50 μM) were analyzed using a Boyden chamber assay. Invasive cells were fixed, stained, and observed using a light microscope (**C**). The bar graph shows the relative value of invasion (%) normalized to the control (**D**). Data are represented as mean ± S.E.M. Significance was set at *p* < 0.05, which is indicated by an asterisk in the graph.

**Figure 7:**
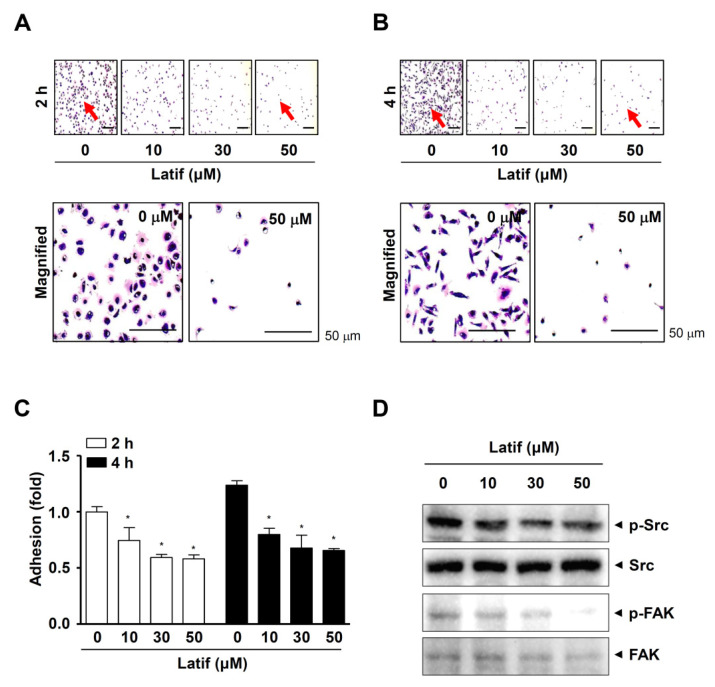
Effects of Latif on cell adhesion and FAK/Src signaling in human YD-10B OSCCs. (**A**–**C**) Latif-treated cells (10, 30, and 50 μM) were seeded onto ECM-coated plates, and then the cell adhesion was monitored using a light microscope at the indicated time points, 2 h (**A**) and 4 h (**B**). Red arrows indicate the magnified region. The bar graph shows the relative value of cell adhesion (fold) normalized to the control (**C**). (**D**) Phospho-Src (p-Src), Src, phospho-FAK (p-FAK), and FAK were analyzed using Western blot analysis. Data are represented as mean ± S.E.M. Significance was set at *p* < 0.05, indicated by an asterisk in the graph.

Since OSCCs have another aggressive feature with frequent infiltration into adjacent tissues, we investigated whether Latif possesses anti-metastatic effects. First, we performed a wound-healing assay to analyze cell mobility. The results showed that Latif significantly blocked the migration of YD-10B OSCCs in a dose-dependent manner after 24 h compared to the control (Figure 6A,B). Second, we performed a Boyden chamber assay to analyze cell penetration through extracellular matrix (ECM) degradation. We found that Latif significantly prevented the invasion of YD-10B OSCCs through the matrigel-coated membrane compared to the control (Figure 6C,D). Third, we performed a cell adhesion assay to analyze cell attachment for matastasis. The results showed that Latif significantly blocked the adhesion of YD-10B OSCCs in a dose- and time-dependent manner after 2 h and 4 h compared to the control, as well as the cellular morphology of the Latif-treated cells was poorly formed despite attachment (Figure 7A–C). In addition, the Western blotting analysis showed that Latif decreased the phosphorylation of non-receptor tyrosine kinase (Src) and focal adhesion kinase (FAK), which are important regulatory proteins in cell metastasis (Figure 7D). 

## 3. Discussion

OSCCs are the most common malignancies in the oral cavity, including the tongue, the gingival, and the lips, which comprise >91% of all oral cancer cases [8,9]. There have been advances in diagnosis and treatment over the past few decades; nevertheless, patients with OSCCs are still life-threatening, with high mortality rates due to aggressive proliferation, high metastasis, and chemotherapeutic resistance [8,11]. Therefore, it is important to improve the outcomes of patients with OSCCs by identifying novel compounds and biological studies in OSCCs. In the present study, we isolated a single compound of Latif (>100% purity) from the heartwood of *D. odorifera*, which has been used as TCM for centuries and investigated anti-cancer activities against OSCCs.

Both benign and malignant tumors occur due to the evasion of cell death in mutant cells [15]. In the present study, we demonstrated that Latif blocks the aggressive proliferation of OSCCs with morphological alteration to a small size with a round shape. Apoptosis is well characterized by the morphological alteration of cell shape and the molecular alteration of nuclear chromatin [16]. Therefore, we first focused on apoptotic cell death and analyzed apoptotic DNA fragmentation in OSCCs. We found that Latif increases DNA fragmentation in OSCCs. It was reported that the apoptotic caspase cascade induces DNA fragmentation by inhibiting inhibitors of caspase-activated DNase, which is a key feature of apoptosis [17,18]. We also found clearer evidence for Latif-induced apoptosis than Latif-induced PARP cleavage products, downregulated Bcl-2 and Survivin protein, and upregulated Bax protein in OSCCs. Previous studies demonstrated that apoptotic caspase cascade is tightly controlled by apoptosis regulator proteins such as Survivin, a member of the inhibitor of apoptosis (IAP), and Bcl-2 family including Bax and Bcl-2 proteins, as well as the PARP cleavage, a hallmark of apoptosis, leads to DNA strand-break-signals and cell cycle arrests [19,20,21]. Moreover, our results demonstrated that Latif downregulates the expression of cell cycle regulatory proteins, including CyclinD1, Cdk4, and Cdk6. Thus, our findings suggest Latif has anti-cancer effects by inducing cell death through apoptotic caspase cascade in OSCCs. 

A more significant number of cases in patients with OSCCs have revealed an alteration in the AKT pathway [22]. AKT is a key regulator of cell survival and proliferation and suppresses apoptotic cell death proteins. One study, in particular, has reported that inhibiting the AKT pathway induces apoptotic cell death in OSCCs [23]. In the present study, we demonstrated that Latif inactivates constitutively active AKT, its upstream protein PI3K, and downstream protein mTOR in OSCCs. Subsequently, we found that Latif inhibits the phosphorylation of p70S6K. It was reported that the increased activity of 70S6K is required for high levels of protein synthesis to enter the cell cycle during cell division, which is a potential target for therapeutic strategies to prevent or inhibit cancer progression [24,25]. Our immunofluorescence observations also found that Latif reduces actively dividing cells, consistent with the dephosphorylation of p70S6K in OSCCs. Thus, our results suggest that Latif regulates cell survival and death through PI3K/AKT/mTOR/p70S6K signaling in OSCCs. 

Although apoptosis is best known to trigger cell death signals, other recent modes of cell death signals include autophagy and necroptosis. Autophagy is a self-degradative process in maintaining homeostasis by either destroying or recycling cellular components, which play critical roles in cell survival and death [26]. Accumulating evidence suggests that autophagy also crosstalks with apoptosis in the occurrence and development of tumor [27,28,29]. MAPKs have been reported as key regulators between autophagy and apoptosis [30]. Our study found that Latif reduces the molecular machinery of autophagy and consequently induces a decrease in autophagosome formation, suggesting that Latif promotes apoptotic cell death by inhibiting autophagy in OSCCs. 

In contrast to apoptosis, necroptosis is a programmed cell death characterized by caspase- independence and is generally regarded as “fail-safe” cell death machinery that occurs when damaged cells cannot induce apoptosis, or the apoptotic mediators are blocked by pharmacological inhibition and genetic mutation [31]. The present study also demonstrated that Latif inhibits the phosphorylation of RIP1, RIP3, and MLKL. Activating RIP1, RIP3, and MLKL is an important molecular event of necroptosis, leading to necrosome formation, MLKL oligomerization, and membrane translocation [32]. It is well known that inhibition of the caspase-8-dependent apoptotic pathway converts apoptosis into necroptosis mode through the activation of molecular events in necroptosis [33]. Thus, our findings suggest that Latif has anti-cancer effects through apoptotic cell death by inhibiting autophagy and necroptosis in OSCCs.

The metastatic process is influenced by the ability of malignancies to evade apoptosis because apoptosis plays an inhibitory function during metastasis [34]. In the present study, we further analyzed the anti-metastasis effect of Latif, demonstrating that Latif has anti-migration and anti-invasion effects in OSCCs. Moreover, we demonstrated that Latif inhibits the adhesion of OSCCs on the ECM and decreases the phosphorylation of FAK and Src. It is important that cell adhesion contributes to metastatic colonization by circulating tumor cells and settling in new niches [35]. The cell adhesion on the ECM induces FAK/Src signaling that regulates the cell shape and migration [36]. In addition to the role of cell adhesion during cell migration and invasion, cell adhesion-induced FAK-Src signaling regulates cell proliferation, survival, and angiogenesis during tumor progression through the various signaling pathways, including activating the AKT pathway [37]. Given that apoptosis also serves as suppressive signals in the metastasis process of malignancies [34], the present data suggest that Latif is a potential compound to prevent metastatic events along with apoptotic cell death by suppressing the AKT pathway in OSCCs. 

## 4. Materials and Methods

### 4.1. General

NMR spectra were obtained on a JEOL ECX-500 spectrometer (JEOL Ltd., Tokyo, Japan) operating at ^1^H-NMR (500 MHz) and ^13^C-NMR (125 MHz) with tetramethylsilane (TMS) as internal standard. High-performance liquid chromatography (HPLC) was performed using Agilent 1260 series (Agilent Technologies, Santa Clara, CA, USA). Column chromatography was conducted using silica gel 60 (70–230 mesh/230–400 mesh ASTM, Merck, Darmstadt, Germany).

### 4.2. Plant Material

The dry aboveground part of *D. odorifera* was purchased at the commercial herbal medicine market. A voucher specimen (P1,350) has been deposited in the Natural Products Bank, National Institute for Korean Medicine Development (NIKOM).

### 4.3. Latif Stock and Sovlent

The Latif was dissolved in 100% dimethyl sulfoxide (DMSO), and the final concentration of 0.1% DMSO was used as the control.

### 4.4. Cell Culture

Human YD-8 and YD-10B OSCCs were purchased from the Korean Cell Line Bank (Seoul, Korea). They were incubated in a humidified atmosphere of 37 °C and 5% CO_2_ using Dulbecco’s Modified Eagle Medium supplemented with heat-inactivated 10% fetal bovine serum and 1 X Gibco^®^ Antibiotic-Antimycotic (Thermo Fisher Scientific, Waltham, MA, USA). 

### 4.5. 3-[4,5-Dimethylthiazol-2-yl]-2,5-diphenyltetrazolium Bromide (MTT) Assay

Cells were seeded onto 96-well plates for 24 h and incubated with Latif for 24 h. MTT assay was performed as previously described [38]. Briefly, MTT solution (5 mg/mL) was added directly to the cells and incubated for 2 h. Formazan was solubilized in 100% DMSO (Sigma-Aldrich, St. Louis, MO, USA), and absorbance was measured at a wavelength of 540 nm using the Multiskan GO Microplate Spectrophotometer (Thermo Fisher Scientific, Waltham, MA, USA).

### 4.6. Cell Migration Assay

Cells were seeded onto 6-well plates and allowed to attach for 24 h. The cells were scratched in the monolayer using a 200 μL pipette tip and incubated in a humidified atmosphere of 37 °C and 5% CO_2_ with Latif. After 24 h, cell migration to the wounded region was monitored using light microscopy.

### 4.7. Cell Invasion Assay

Boyden chamber assay was performed to investigate the cell invasion rate as previously described [39]. Briefly, 2 × 10^4^ cells were seeded onto nucleopore filters coated with matrigel solution (Corning Life Sciences, Tewksbury, MA, USA) and allowed to infiltrate for 4 h in a humidified atmosphere of 37 °C and 5% CO_2_. The cell invasion was monitored using light microscopy.

### 4.8. Cell Adhesion Assay

Matrigel solution (Corning Life Sciences) was added onto a 96-well culture plate and solidified for 1 h. 1 × 10^4^ cells were seeded in the 96-well plate and allowed to attach for 2 h and 4 h in a humidified atmosphere of 37 °C and 5% CO_2_. The cells were fixed in 10% formalin for 10 min, stained with 0.5% crystal violet for 10 min, and monitored using light microscopy. To quantify the adhesive cells, the stains were solubilized in 100% DMSO (Sigma-Aldrich), and absorbance was measured at a wavelength of 595 nm using the Multiskan GO Microplate Spectrophotometer (Thermo Fisher Scientific).

### 4.9. Terminal Deoxynucleotidyl Transferase-Mediated FITC–dUDP Nick-End Labeling (TUNEL) Assay

In situ Cell Death Detection Kit (Roche Diagnostics GmbH, Mannheim, Germany) was used to monitor apoptotic DNA fragmentation as previously described [39]. Briefly, cells were incubated in a reaction mix containing deoxynucleotidyl transferase and FITC–dUDP for 1 h at 37 °C in the dark and then counterstained with 4′,6′-diamidino-2-phenylindole dihydrochloride (DAPI, Sigma) solution for 10 min. The slides were mounted and apoptotic DNA fragmentation was observed through a fluorescence microscope.

### 4.10. Western Blot Analysis

Western blot analysis was carried out as previously described [40,41]. Briefly, protein concentration was determined using Bradford reagent (Bio-Rad, Hercules, CA, USA), and 20 μg proteins were subjected to sodium dodecyl-polyacrylamide gel electrophoresis and then transferred to PVDF membranes, which were blocked using 5% skim milk for 1 h. The membranes were incubated overnight at 4 °C with the specific primary antibodies, washed three times, and incubated with the secondary antibodies (1:10,000, Jackson ImmunoResearch, West Grove, PA, USA) for 1 h at room temperature. After three washes, protein signals were detected using an enhanced chemiluminescence Kit (Millipore, Bedford, MA, USA) in the ProteinSimple detection system (ProteinSimple Inc., Santa Clara, CA, USA).

### 4.11. Immunofluorescence Assay

Immunofluorescence assay was accessed as previously described [39,42]. Briefly, Cells were fixed, permeabilized, washed, and blocked using 3% BSA for 1 h at room temperature. The specific primary antibodies were incubated overnight at 4 °C, washed three times, and incubated with the secondary antibodies (Invitrogen, Carlsbad, CA, USA) for 2 h at room temperature in the dark. After that, the cells were counterstained with DAPI (Sigma) solution for 10 min in the dark. The slides were mounted, and immunofluorescence was observed through a fluorescence microscope or the intravital multi-photon microscope system (IMPM) at Gwangju Center, Korea Basic Science Institute (KBSI).

### 4.12. Autophagy Detection Assay

DAPGreen Autophagy Detection Kit (Dojindo, Japan) is used to monitor autophagic vacuole formation according to the manufacturer’s instructions. Briefly, cells were washed in culture medium twice, and DAPGreen solution (0.1 μM) was added to the cells for 30 min. After that, the cells were washed in a culture medium twice and incubated with Latif for 6 h. The cells were fixed in 10% formalin, the slides were mounted, and then autophagic vacuole formation was observed through a fluorescence microscope.

### 4.13. Statistical Analysis

Data were analyzed using the GraphPad Prism version 5 program (GraphPad Software, Inc., San Diego, CA, USA). All numeric values are represented as mean ± S.E.M. Statistical significance was assessed through the Student’s unpaired *t*-test. Significance was set at *p* < 0.05, indicated by an asterisk in the graph.

## 5. Conclusions

In conclusion, our study is the first to provide new evidence that Latif promotes apoptotic cell death through the inhibition of the AKT pathway, thereby blocking cell growth, division, migration, invasion, and adhesion of OSCCs. Currently, epidermal growth factor receptor inhibitors and cetuximab have been used as commercially available targeted therapies for patients with OSCCs [14]. Thus, our data suggest that Latif may be an important therapeutic compound and help prevent and treat patients with OSCCs.

## Data Availability

The data that support the findings of this study are available from the corresponding author upon reasonable request.

## Supplementary Materials

The supporting information can be downloaded at: https://www.mdpi.com/article/10.3390/ijms232113629/s1.

## Abbreviations

Caspases	cysteinyl-aspartate proteases
ECM	Extracellular matrix
FAK	Focal adhesion kinase
Src	Non-receptor tyrosine kinase
HPLC	High performance liquid chromatography
MAPK	Mitogen-activated protein kinases
MTT	3-[4,5-dimethylthiazol-2-yl]-2,5-diphenyltetrazolium bromide
PARP	Poly ADP-ribose polymerase
OSCC	Oral squamous cell carcinoma
