# Supplementary material for: Latifolin, a Natural Flavonoid, Isolated from the Heartwood of Dalbergia odorifera Induces Bioactivities through Apoptosis, Autophagy, and Necroptosis in Human Oral Squamous Cell Carcinoma
Source: Int J Mol Sci. 2022 Nov 7;23(21):13629. doi: 10.3390/ijms232113629 (PMC9655104; doi:10.3390/ijms232113629)
Supplement: Supplementary file 1 [file ijms-23-13629-s001.zip › ijms-1978384-supplementary.pdf]

Supplementary Figure Legend

- **Supplementary Figure S1. NMR of Latif from heartwood of *D. odorifera.***
- **(A, B)** The ^{1}H-NMR (500 MHz, CD_3OD) spectrum (A) and ^{13}C-NMR (125 MHz, CD_3OD) spectrum (B) of Latif

A

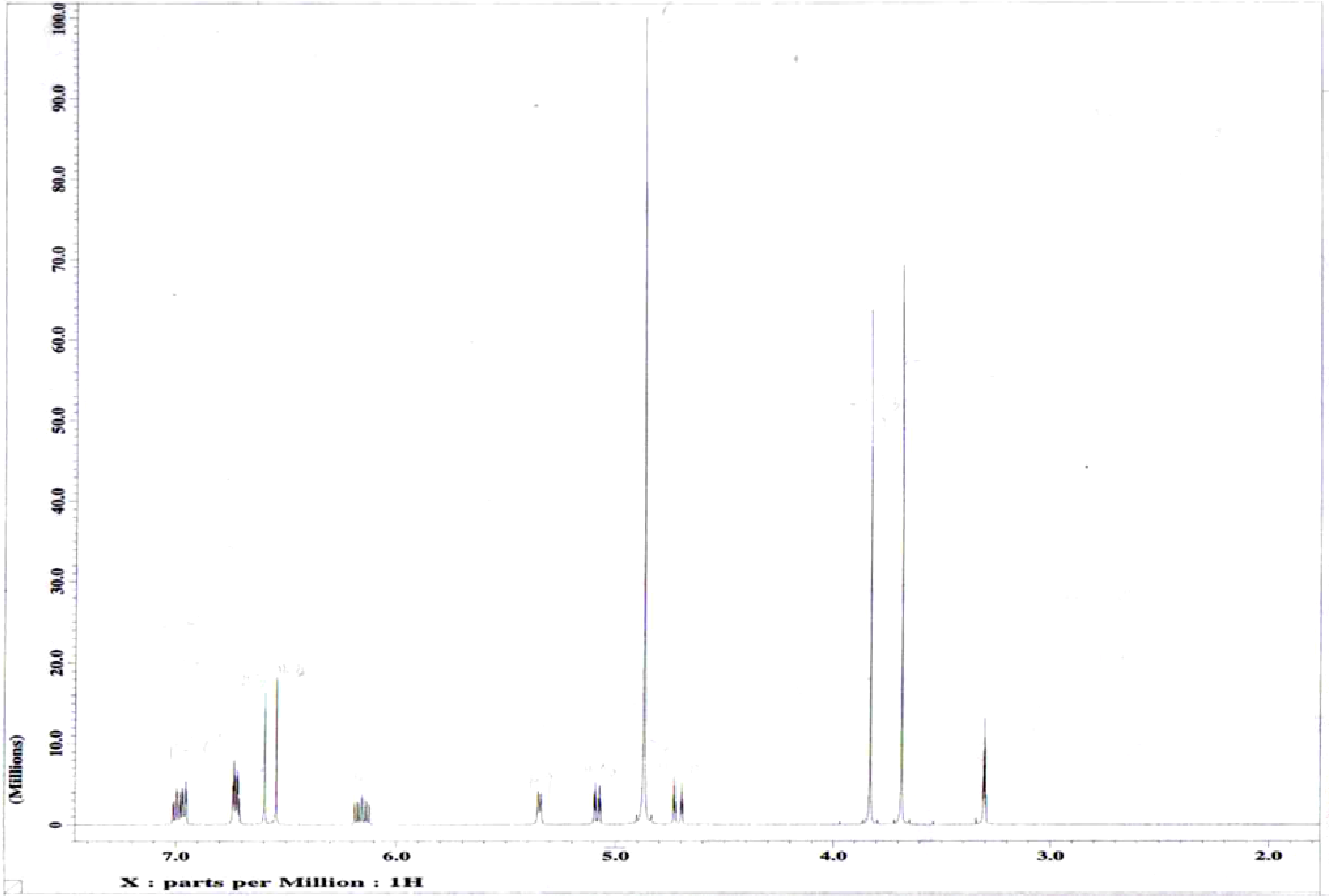

(Millions)
0
10.0
20.0
30.0
40.0
50.0
60.0
70.0
80.0
90.0
100.0
7.0
6.0
5.0
4.0
3.0
2.0
X : parts per Million : 1H

B

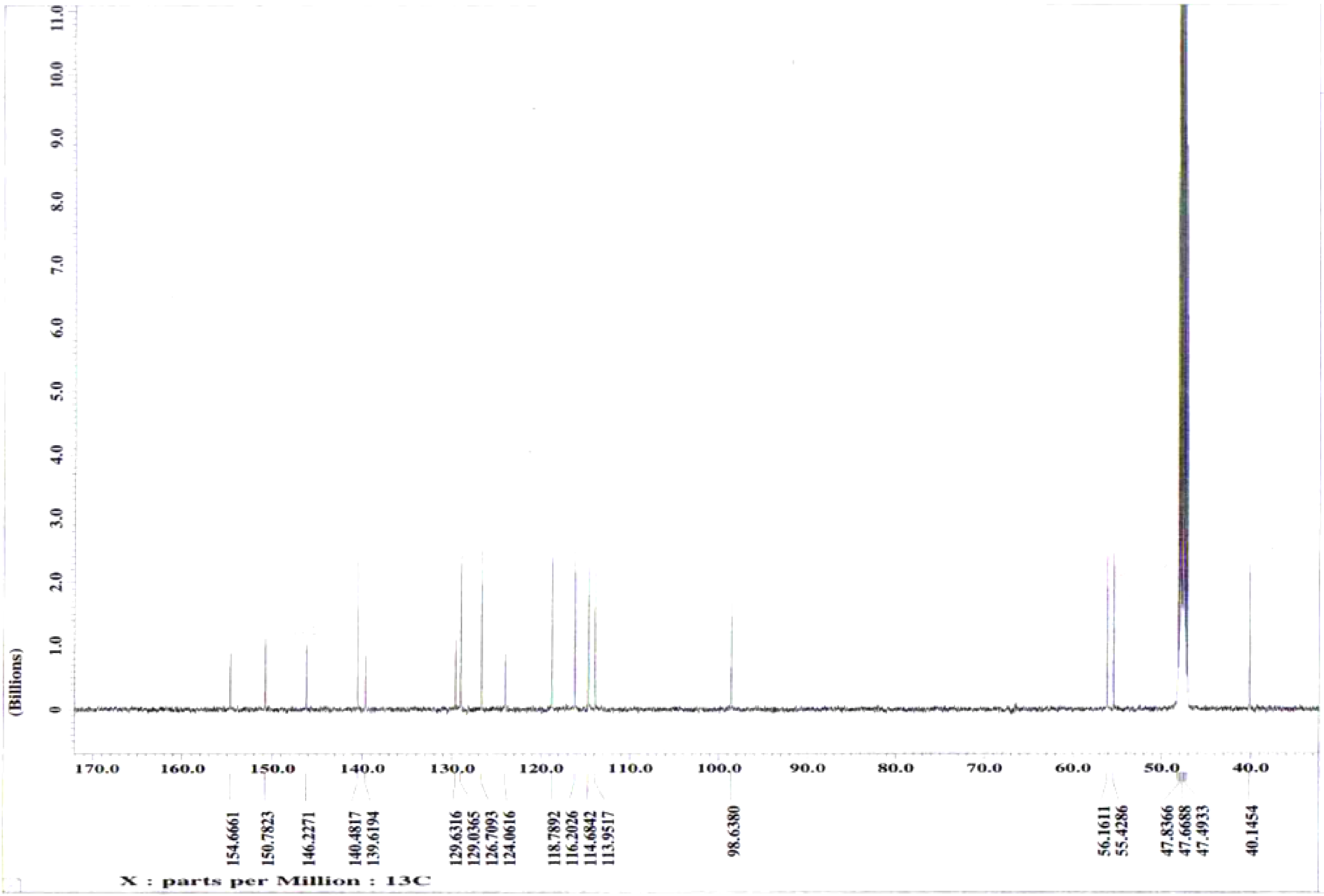
(Billions)
11.0
10.0
9.0
8.0
7.0
6.0
5.0
4.0
3.0
2.0
1.0
0
170.0 160.0 150.0 140.0 130.0 120.0 110.0 100.0 90.0 80.0 70.0 60.0 50.0 40.0
154.6661
150.7823
146.2271
140.4817
139.6194
129.6316
129.0365
126.7093
124.0616
118.7892
116.2026
114.6842
113.9517
98.6380
56.1611
55.4286
47.8366
47.6688
47.4933
40.1454
X : parts per Million : 13C
